# Lymphedema as an initial manifestation of paraneoplastic rheumatic syndromes: a case series of six patients

**DOI:** 10.3389/fonc.2026.1964817

**Published:** 2026-10-26

**Authors:** Guo-Hua Zhang, Ling-Ling Zhang, Min-Na Jiang, Wei-Xia Wang, Kai-Li Zhong, Yu-Hua Wang

**Affiliations:** 1 Department of Rheumatology and Clinical Immunology, Beijing Shijitan Hospital, Capital Medical University, Beijing, China; 2 Department of Lymphoma, Beijing Shijitan Hospital, Capital Medical University, Beijing, China

**Keywords:** early detection, lymphedema, lymphoma, malignancy, paraneoplastic rheumatic syndromes

## Abstract

**Objective:**

Lymphedema as the initial symptom poses significant challenges in the early diagnosis of underlying malignancies masquerading as rheumatic diseases.

**Methods:**

This study retrospectively analyzed six patients who were hospitalized at Beijing Shijitan Hospital between October 2015 and October 2025 with paraneoplastic rheumatic syndromes (PRS) presenting with lymphedema as the initial manifestation. Data on clinical manifestation, laboratory and imaging findings, diagnoses, treatments, and prognosis were collected.

**Results:**

Six patients (3 males, 3 females; median age 66 years) presented with lymphedema as the initial manifestation of PRS. The initial rheumatologic diagnoses were leukocytoclastic vasculitis (n=2), undifferentiated connective tissue disease (n=2), Sjögren’s syndrome (n=1), and anti-synthetase syndrome (n=1). The median interval from lymphedema onset to rheumatic diagnosis was 5.5 months. All patients received glucocorticoids, and four received immunosuppressants; none achieved durable benefit. The median interval from rheumatic diagnosis to therapy failure was 1 month, and from therapy failure to cancer diagnosis was 1 month. The underlying malignancies included lymphoma (n=3), lung adenocarcinoma (n=2), and gastric adenocarcinoma (n=1). During a follow-up of 1 to 13 months, two patients showed significant improvement in lymphedema, whereas the remaining four died.

**Conclusion:**

Malignancies may present with lymphedema as the initial symptom, mimicking rheumatic diseases. Underlying malignancy should be highly suspected, particularly when there is a poor response to immunosuppressive therapy. Early recognition and screening for malignancies are crucial for improving patient outcomes.

## Introduction

Paraneoplastic rheumatic syndromes (PRS) refer to a condition in which occult malignancy mimics rheumatic diseases through humoral or cellular immune mechanisms distinct from direct tumor invasion or metastasis (1). Accumulating evidence has established a significant association between rheumatic manifestations and underlying neoplasms, encompassing conditions such as paraneoplastic polyarthritis, cancer-associated myositis (CAM), and paraneoplastic vasculitis (2–4). However, due to the often clinically silent nature of the primary tumors, early recognition of PRS remains a substantial diagnostic challenge, frequently leading to delayed diagnosis and unfavorable clinical outcomes.

Lymphedema results from impaired lymphatic circulation and persistent accumulation of protein-rich interstitial fluid, commonly affecting the lower limbs, upper arms, genitals, and face. It is classified as primary or secondary. Primary lymphedema is mainly caused by genetic factors, whereas secondary lymphedema arises from lymphatic injury due to infection, trauma, cancer therapy, or chronic lymphatic overload (5, 6). While rheumatic diseases such as systemic lupus erythematosus and rheumatoid arthritis can occasionally involve the lymphatic system and manifest as lymphedema (7, 8), such presentations are rare and documented only in limited case series. To the best of our knowledge, no previous study has reported lymphedema as the initial symptom of PRS.

Given this diagnostic gap, we conducted a retrospective analysis of six patients who presented with lymphedema as the initial manifestation of an underlying malignancy. This case series aims to underscore the critical need to consider PRS in patients with unexplained lymphedema and atypical rheumatologic features, particularly when the disease proves refractory to conventional immunosuppressive therapies.

## Methods

### Study population

We retrospectively enrolled six patients who presented with lymphedema as the initial manifestation of PRS at Beijing Shijitan Hospital, Capital Medical University, between October 2015 and October 2025.

### Diagnostic criteria and approach

The group comprised two cases of leukocytoclastic vasculitis, two cases of undifferentiated connective tissue disease (UCTD), one case of Sjögren’s syndrome (SS), and one case of anti-synthetase syndrome (ASS). Diagnosis was based on the following criteria: (1) leukocytoclastic vasculitis, confirmed by skin biopsy; (2) the 2002 International Classification Criteria for SS; (3) the Connors criteria for ASS, including positive serum anti-aminoacyl-tRNA synthetase (anti-ARS) antibodies combined with one or more of the following manifestations—PM/DM (Bohan/Peter criteria), ILD (ATS criteria), arthritis, fever, Raynaud’s phenomenon, and mechanic’s hands; and (4) Corte and colleagues’ criteria for UCTD.

The diagnosis of malignancy was confirmed by histopathology. None of the patients had a personal or family history of malignancy. lymphedema was confirmed by lymphoscintigraphy in all patients, and other causes of limb edema, such as venous thrombosis, cardiac, renal, or hepatic insufficiency, and hypoalbuminemia, were excluded. Mechanical causes of lymphedema, such as tumor invasion, nodal compression, thoracic duct involvement, intravascular tumor occlusion, and treatment-related lymphatic injury, were also excluded by imaging and clinical review. In the absence of internationally accepted diagnostic criteria for PRS, we applied the diagnostic approach proposed by Cho et al. (9). After excluding direct tumor invasion, metastasis, and treatment-related factors, the diagnosis of PRS was based on a comprehensive assessment of temporal association, atypical clinical features, response to conventional antirheumatic therapy, and outcome following tumor treatment.

### Data collection

Demographic data, clinical manifestations, physical examination findings, and malignancy history were extracted from medical records. Laboratory findings, including the level of erythrocyte sedimentation rate (ESR) (>20 mm/h), the level of C-reactive protein (CRP) (>5 mg/L), anti-nuclear antibody (ANA), anti-extractable nuclear antigen (anti-ENA) antibody, rheumatoid factor (RF), and anti-neutrophil cytoplasmic antibody (ANCA), were evaluated at diagnosis. The diagnosis of malignancy was confirmed in all patients by pathological examination. Ethical approval was granted by the Ethics Committee of Beijing Shijitan Hospital, Capital Medical University (sjtkyll-jy-2023(006)), with a waiver of informed consent due to its retrospective nature.

### Statistical analysis

All data processing and statistical analyses were performed using SPSS software (version 23.0, IBM, Armonk, NY). All data were analyzed and presented as median (range).

## Results

### Demographic characteristics

The demographics of all patients are summarized in **Table 1**. The cohort included three males and three females. The median age at lymphedema onset was 66 years (range, 57–74 years). The median time interval from lymphedema onset to rheumatic diagnosis was 5.5 months (range, 1–22 months).

**Table 1:** Demographics, diagnosis, treatment and prognosis of PRS patients with lymphedema as the initial manifestation.

Case	Sex	Age of LE onset years	TI: LE onset to RD months	RD	Rheumatism Treatment	TI: RD to IS failure months	Malignancy	TI: IS failure to cancer diagnosis months	Malignancy treatment	Follow-up duration (months)	Prognosis
1	F	74	11	LV	GC	0.5	Intravascular Large B-cell Lymphoma	0.5	R-CHOP	10	Lymphedema improved significantly.
2	F	57	1	SS	GC+HCQ+CTX	1	Gastric adenocarcinoma.	0.5	oral S-1	2	death
3	M	68	1	ASS	GC+CsA	1	Peripheral T-cell lymphoma	1	None	1	death
4	F	64	3	UCTD	GC+HCQ	5	Lung adenocarcinoma	1	surgery immunotherapy	13	death
5	M	67	22	LV	GC	1	NK/T cell lymphoma	1	L-GDP	3	death
6	M	64	8	UCTD	GC+MMF	9	Lung adenocarcinoma	1.5	surgery	11	Lymphedema improved significantly.

*LE*, lymphedema*; TI*, Time interval*; RD*, Rheumatism diagnosis; *LV*, leukocytoclastic vasculitis; *UCTD*, undifferentiated connective tissue disorder; *SS*, sjogren’s syndrome, *ASS*, anti-synthetase syndrome; *GC*, glucocorticoid; *HCQ*, hydroxychloroquine; *CTX*, cyclophosphamide, *MMF*, mycophenolate mofetil; *CsA*, cyclosporine; IS *Immunosuppressants, R-CHOP*, (Rituximab, Cyclophosphamide, Doxorubicin, Vincristine, and prednisone); *L-GDP*, (Gemcitabine, Dexamethasone, Cisplatin).

### Clinical features

All six patients presented with lymphedema as their initial symptom. Specifically, three patients had bilateral lower limb lymphedema, two had bilateral upper and lower limb involvement, and one had bilateral upper limb and facial lymphedema. Associated rheumatologic findings included cutaneous involvement in five patients (skin rash in two, erythema nodosum in one, erythema and ulceration in one, and Gottron’s sign in one), arthritis in two patients, protein-losing enteropathy (PLE) in two patients, lymphadenopathy in two patients, fever in two patients, chylous ascites in one patient, and Raynaud’s phenomenon in one patient (**Table 2**).

**Table 2:** Clinical characteristics and laboratory findings of PRS patients with lymphedema as the initial manifestation.

Case	Clinical Characteristics		ESR mm/hr	CRP mg/dl	RF	ANA	Anti-ENA	ANCA
	lymphedema	Other symptoms						
1	bilateral upper/lower limbs	fever erythema nodosum	45	53.8	N	- N	- N	- N
2	bilateral lower limbs	chylous ascites dry mouth skin rash PLE	38	33.6	N	S1:320	SSA/RO52	N.
3	bilateral lower limbs	Gottron’s sign Raynaud’s phenomenon ILD PLE lymphadenopathy	19	9.9	N	N	N	N
4	bilateral upper limbs and facial	arthritis	17	10.6	N	HS1:320	RO52	N
5	bilateral upper/lower limbs	erythema and ulcer lymphadenopathy fever	44	11.4	N	N	N	N
6	bilateral lower limbs	skin rash arthritis	38	18.42	N	S1:100	N	N

*ILD*, Interstitial Lung Disease; *PLE*, Protein-losing Enteropathy*; ANA*, anti-nuclear antibody; *anti-ENA antibody* anti-extractable nuclear antigen antibody; *RF*, rheumatoid factor; *ANCA*, anti-neutrophil cytoplasmic antibody; *N*, negative.

### Laboratory features

At the diagnosis of rheumatic diseases, the median CRP level was 14.9 mg/L (range, 9.9–53.9 mg/L) and the median ESR level was 38 mm/h (range, 17–45 mm/h). CRP was elevated in all six patients, and ESR was elevated in four. ANA positivity was observed in three patients, while two were positive for anti-ENA antibodies (one with anti-SSA/Ro52 and one with anti-Ro52 alone). One patient tested positive for anti-YARS antibodies (a myositis-specific antibody), whereas RF and ANCA were negative in all cases.

Lymphoscintigraphy, performed in all patients, confirmed bilateral lower limb and/or upper and/or facial lymphedema (**Figure 1**). Protein-losing imaging scintigraphy, performed in two patients, indicated intestinal protein loss in the ileum (Case 2, **Figure 2**) and ascending colon (Case 3, **Figure 3**). PET-CT scans were performed in five patients. Two of these patients showed multiple lymphadenopathies. PET-CT in these two patients showed no mesenteric/retroperitoneal nodal compression, thoracic duct obstruction, or intravascular tumor occlusion. The patient with chylous ascites had no thoracic duct obstruction or mass-effect chylous leak; the patient with IVLBCL had no radiologic or histopathologic large-vessel or lymphatic obstruction. No treatment-related lymphatic injury was identified, and no patient was excluded from the PRS classification for confirmed mechanical obstruction.

**Figure 1:**
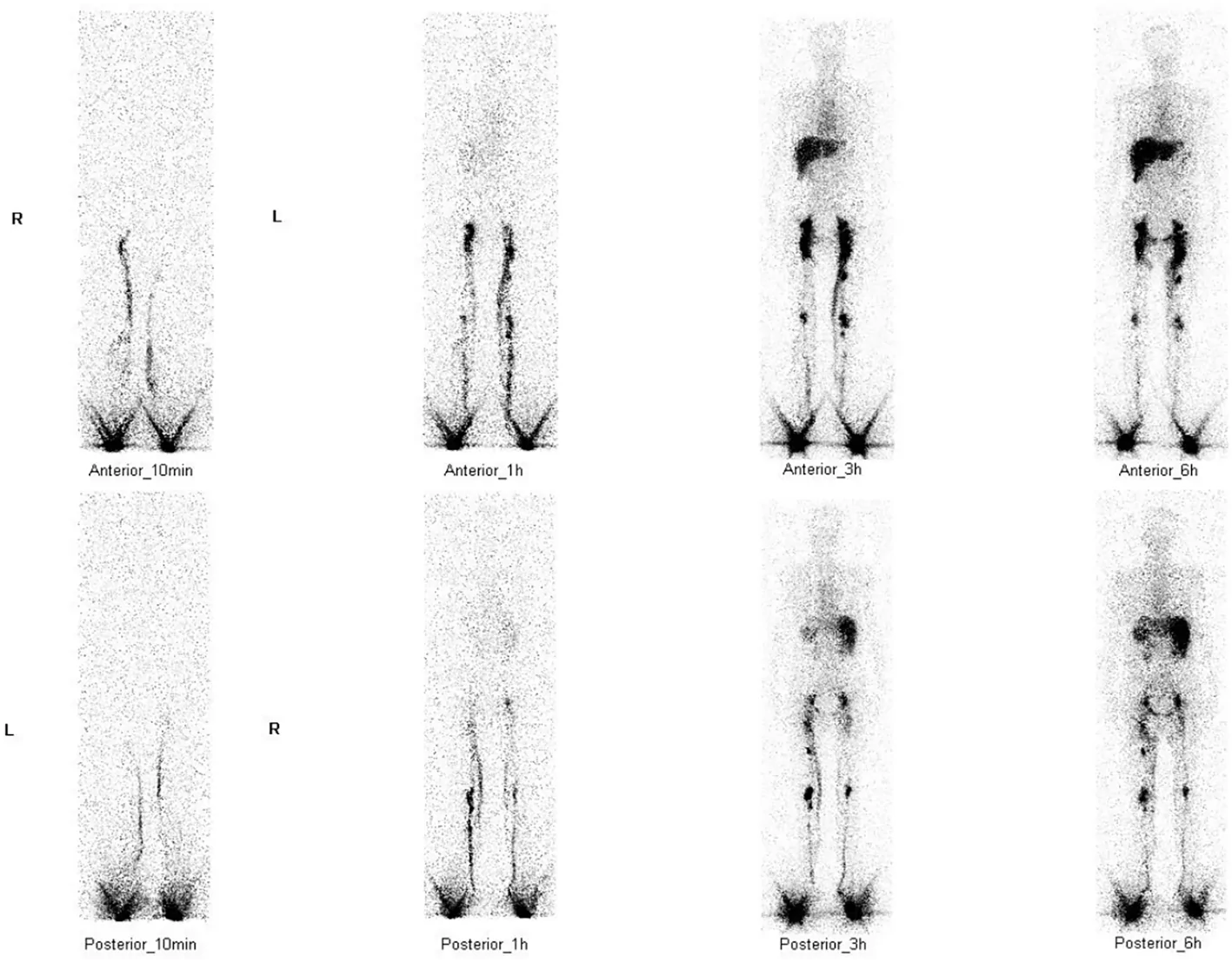
Lymphoscintigraphy suggested lymphedema in the left lower limb and chylous ascites.

**Figure 2:**
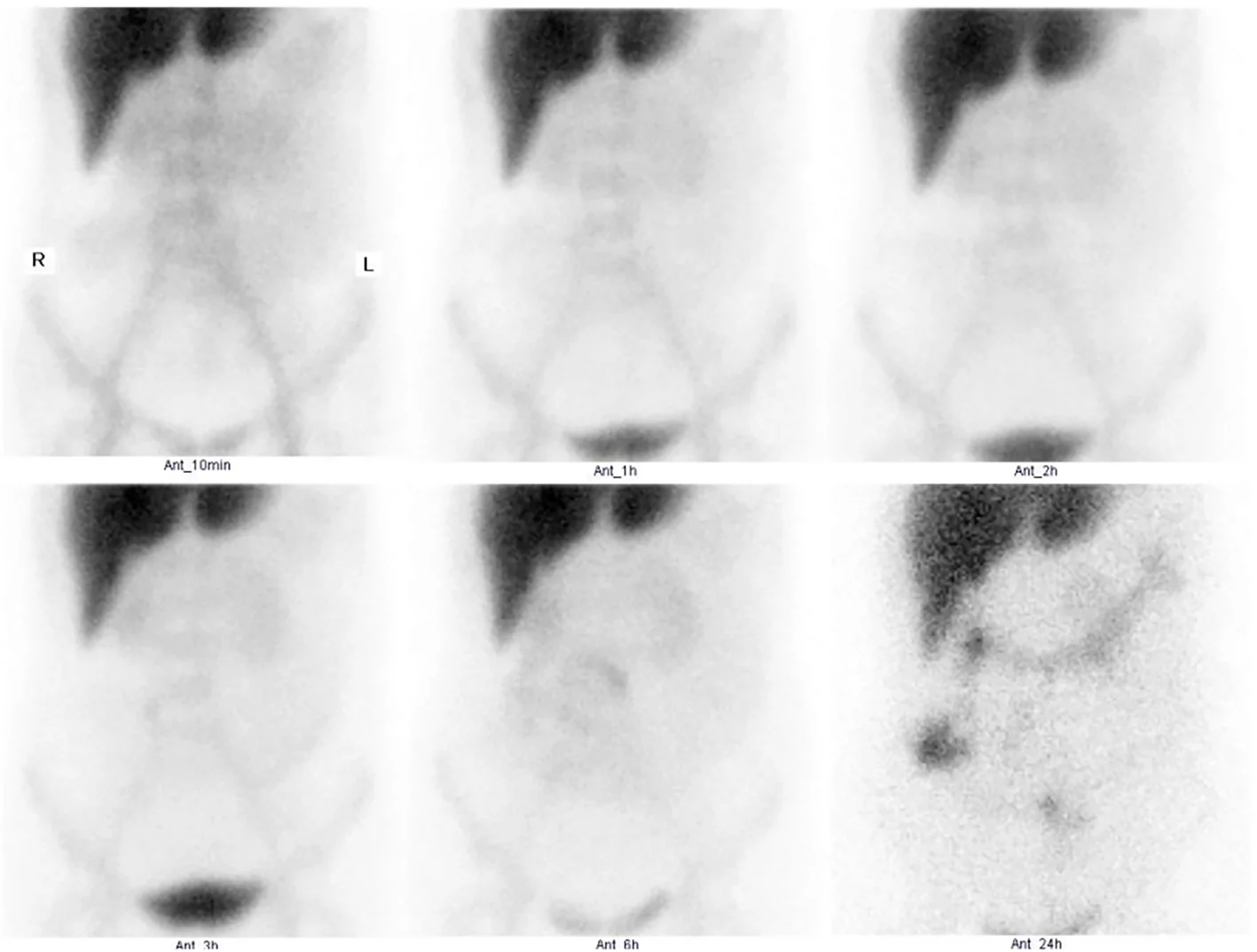
Protein-losing imaging indicated intestinal protein loss (ileum).

**Figure 3:**
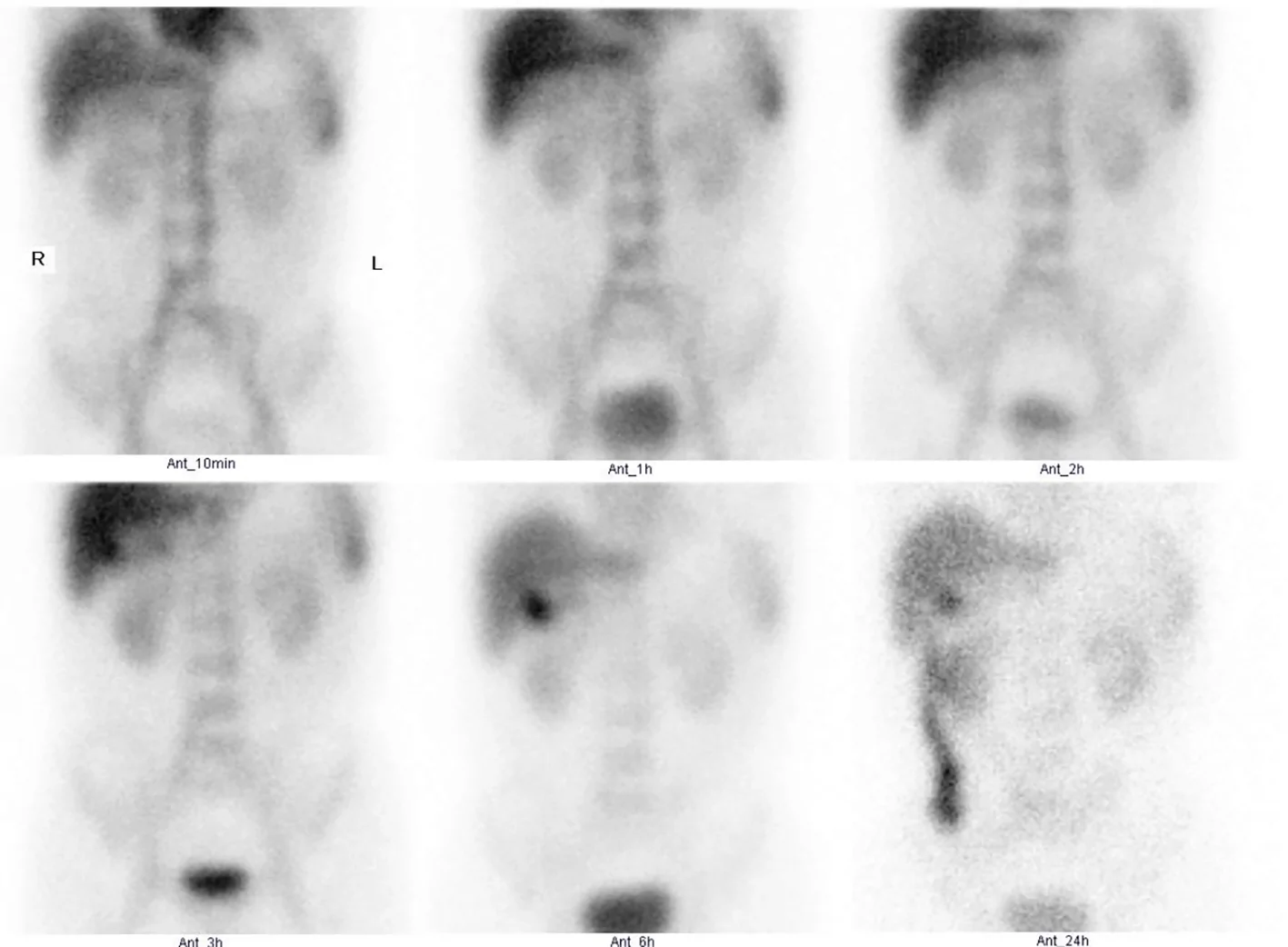
Protein-losing imaging indicated protein loss (the ascending colon).

### Spectrum of rheumatic diseases and underlying malignancies

The median time interval from rheumatism diagnosis to failure of glucocorticoid and/or immunosuppressant therapy was 1 month (range, 0.5–9 months). Therapy failure was defined as lack of clinical improvement or disease progression despite adequate glucocorticoid and/or immunosuppressant therapy. The median time interval from therapy failure to cancer diagnosis was 1 month (range, 0.5–1.5 months). Among the six patients, three were diagnosed with solid tumors, including lung adenocarcinoma (n=2) and gastric adenocarcinoma (n=1), and three with lymphoma, including intravascular large B-cell lymphoma (IVLBCL) (n=1), peripheral T-cell lymphoma (n=1), and NK/T cell lymphoma (n=1). Notably, the two patients with initial leukocytoclastic vasculitis were ultimately diagnosed with lymphoma. Although ASS was supported by muscle biopsy in one case, the patient was ultimately diagnosed with lymphoma (**Table 1**).

### Treatment and prognosis

All six patients received glucocorticoid therapy for their rheumatic disease, and four also received immunosuppressants (hydroxychloroquine in two; mycophenolate mofetil, cyclophosphamide, and cyclosporine in one each). None achieved durable benefit: four patients showed suboptimal responses, and two patients initially responded but experienced disease progression during subsequent therapy. The median interval from rheumatologic diagnosis to treatment failure was 1 month (range, 0.5–9 months).

Malignancy-directed treatments varied: Case 1 (IVLBCL): treated with six cycles of R-CHOP (Rituximab, Cyclophosphamide, Doxorubicin, Vincristine, and prednisone) chemotherapy, resulting in complete resolution of limb lymphedema. Case 2 (gastric adenocarcinoma): received oral S-1 but progressed, and the patient succumbed to the disease. Case 3 (peripheral T-cell lymphoma): did not receive chemotherapy due to clinical deterioration and died. Case 4 (lung adenocarcinoma): treated with surgery and immunotherapy but progressed, and the patient died. Case 5 (NK/T-cell lymphoma): received two cycles of L-GDP (Gemcitabine, Dexamethasone, Cisplatin) chemotherapy but ultimately died from disease progression. Case 6 (lung adenocarcinoma): treated with surgery alone, resulting in marked improvement in limb lymphedema (**Table 1**).

During a follow-up of 1 to 13 months, two patients showed significant improvement in lymphedema, whereas the remaining four died (**Table 1**).

## Discussion

### Lymphedema as a sentinel manifestation of paraneoplastic rheumatic syndromes

Lymphedema arises from structural or functional disruption of the lymphatic system, leading to distal lymphatic fluid stasis and interstitial accumulation (6). Radionuclide lymphoscintigraphy or lymphangiography serves as diagnostic tools for lymphedema and chylous effusions (10, 11). These imaging techniques reveal structural changes in the lymphatic system and dynamically display lymphatic transport function, helping to identify etiology, select surgical candidates, and monitor treatment efficacy (11, 12). All six patients in our study had lymphoscintigraphic findings consistent with lymphedema.

While secondary lymphedema is commonly attributed to surgery, trauma, infection, or oncologic treatments, our series highlights a less recognized cause: paraneoplastic rheumatic syndromes. The rarity of lymphatic involvement in connective tissue diseases is well recognized. For instance, Zhang et al. reported that systemic lupus erythematosus can, albeit rarely, lead to lymphedema through lymphatic vessel involvement (7). Consistent with this, Yang et al. demonstrated that while lymphatic system involvement is detectable by advanced imaging in systemic lupus erythematosus and rheumatoid arthritis, clinically apparent lymphedema is exceptionally uncommon, predominantly manifesting as chylous effusions or subclinical lymphatic dysfunction (8). However, our findings extend this observation, suggesting that lymphedema itself may be a sentinel manifestation of an underlying paraneoplastic process, rather than solely a complication of a primary rheumatic disease or its treatment.

In our cohort, all patients initially met criteria for rheumatologic conditions, including leukocytoclastic vasculitis, UCTD, SS, and ASS. The subsequent discovery of malignancies—including lymphomas and adenocarcinomas—underscores the clinical overlap between PRS and primary rheumatic disorders. This diagnostic mimicry is particularly well documented for paraneoplastic vasculitis (4, 13, 14), where leukocytoclastic vasculitis is the most common phenotype, frequently associated with both solid and hematologic malignancies (13, 14). Similarly, patients with SS and UCTD in our cohort were ultimately found to have gastric and lung cancers, aligning with previous reports linking these conditions to lymphoma and solid tumors (4).

The high index of suspicion for malignancy in PRS is further supported by the lack of durable response to immunosuppressive therapy observed in all six patients, which was especially notable in the case with an initial diagnosis of ASS. Unlike dermatomyositis, where anti-TIF1-γ antibodies serve as robust biomarkers for cancer-associated myositis (15), the association between ASS and malignancy remains controversial. Anti-Jo1 antibodies have even been suggested to be protective (16), while anti-OJ antibodies may correlate with higher cancer risk (17). In our study, the ASS patient positive for anti-YARS antibodies failed to respond to glucocorticoid and cyclosporine and was subsequently diagnosed with T-cell lymphoma. Whether anti-YARS antibodies herald a paraneoplastic process warrants further investigation.

### Potential immune-mediated mechanisms

In our cohort, we considered lymphedema to be predominantly immune-mediated. Because PRS requires an immune-mediated rheumatic manifestation distinct from direct tumor invasion or metastasis, patients with confirmed mechanical obstruction were not classified as PRS. Mechanical causes—including tumor invasion, nodal compression, thoracic duct involvement, intravascular tumor occlusion, and treatment-related lymphatic injury—were excluded by imaging and clinical review. Although PLE, multiple lymphadenopathies, chylous ascites, and IVLBCL raised concern for mechanical contributors, these were excluded or deemed insufficient to explain the lymphedema. In particular, cross-sectional imaging showed no evidence of mesenteric or retroperitoneal lymph node compression, thoracic duct obstruction, or chylous leak attributable to mass effect, and no radiologic or histopathologic evidence of large-vessel or lymphatic obstruction was found in the patient with IVLBCL.

Two patients had biopsy-proven leukocytoclastic vasculitis, indicating immune-mediated vascular injury that could impair capillary and lymphatic endothelial function and increase vascular permeability. All six patients had elevated CRP and four had elevated ESR, suggesting that systemic immune dysregulation contributed to lymphatic dysfunction. Multi-level lymphatic involvement was also observed: PLE and chylous ascites reflect loss of lymphatic integrity in the intestinal and peritoneal compartments, whereas lymphadenopathies on PET-CT suggest widespread lymphatic activation or tumor infiltration. IVLBCL is especially relevant, as it can cause systemic inflammation and endothelial injury without overt nodal disease, supporting a remote effect of occult tumor on lymphatic function. In this patient, the absence of demonstrable vascular or lymphatic obstruction argues against microvascular tumor embolization as the principal mechanism. This is consistent with the known clinical presentation of IVLBCL, in which fever and systemic inflammatory symptoms are common while lymphadenopathy is typically absent, reflecting the predominantly intravascular nature of the disease (18).

Finally, the temporal sequence—lymphedema onset preceding rheumatic diagnosis, followed by immunosuppressive failure and subsequent cancer diagnosis—is consistent with a paraneoplastic process. The poor response to glucocorticoids and immunosuppressants in all six patients, followed by cancer diagnosis within a median of 1 month, supports the concept that the underlying malignancy was the principal driver of the clinical syndrome. Notably, the two patients who showed significant improvement in lymphedema received effective tumor-directed treatment (R-CHOP and surgery, respectively), suggesting that targeting the tumor may reverse the immune-mediated process responsible for lymphatic dysfunction.

### Limitations

Our study is limited by its retrospective design, small sample size, single-center setting, and absence of a control group. Lymphedema was not systematically staged before lymphoscintigraphy; although lymphoscintigraphy confirmed the diagnosis in all patients, standardized tools such as ISL staging, the Stemmer sign, and limb volume measurement were not consistently applied. Retrospective adjudication relied on available imaging and histopathologic data, microvascular tumor embolization therefore cannot be entirely excluded, particularly in IVLBCL, although the absence of vascular occlusion, the multi-level and multi-compartment involvement, the systemic inflammatory markers, the biopsy-proven leukocytoclastic vasculitis, and the response to tumor-directed therapy favor an immune/cytokine-mediated process. In addition, because of the small number of cases and the lack of a control group, the association between lymphedema and malignancy should be interpreted as an important clinical clue rather than a definitive causal relationship. Future prospective studies with larger cohorts should incorporate standardized lymphedema staging and volumetric assessment, and are needed to elucidate pathophysiology and identify biomarkers predicting malignancy in patients presenting with lymphedema and rheumatic features.

## Clinical implications and conclusion

This series emphasizes two key messages for clinicians. First, lymphedema can be an initial and isolated manifestation of PRS. Second, the lack of response to glucocorticoids and/or immunosuppressants should trigger a comprehensive work-up for occult malignancy, including PET-CT, lymphoscintigraphy, and, when appropriate, biopsy of suspicious lesions. A multidisciplinary approach involving rheumatologists, oncologists, and lymphatic specialists is critical for timely diagnosis and treatment. These findings underscore the importance of recognizing lymphedema as a potential early indicator of paraneoplastic disease. Given the small sample size and retrospective design, these findings should be regarded as a clinical clue rather than proof of a causal association. Clinicians should maintain a high index of suspicion for occult malignancy in patients with atypical rheumatologic symptoms accompanied by lymphedema, especially when there is a poor response to conventional immunosuppressive therapies.

## Data Availability

The raw data supporting the conclusions of this article will be made available by the authors, without undue reservation.

## References

[1] András C Csiki Z Ponyi A Illés A Dankó K . Paraneoplastic rheumatic syndromes. Rheumatol Int. (2006) 26:376–82. doi: 10.1007/s00296-005-0005-3 16091919

[2] Manger B Schett G . Rheumatic paraneoplastic syndromes - a clinical link between malignancy and autoimmunity. Clin Immunol. (2018) 186:67–70. doi: 10.1016/j.clim.2017.07.021 28736272

[3] Turesson C Matteson EL . Malignancy as a comorbidity in rheumatic diseases. Rheumatol (Oxford). (2013) 52:5–14. doi: 10.1093/rheumatology/kes189 22829694

[4] Solans-Laqué R Bosch-Gil JA Pérez-Bocanegra C Selva-O'Callaghan A Simeón-Aznar CP Vilardell-Tarres M . Paraneoplastic vasculitis in patients with solid tumors: report of 15 cases. J Rheumatol. (2008) 35:294–304. 18085729

[5] Brouillard P Boon L Vikkula M . Genetics of lymphatic anomalies. J Clin Invest. (2014) 124:898–904. doi: 10.1172/JCI71614 24590274 PMC3938256

[6] Jiang X Nicolls MR Tian W Rockson SG . Lymphatic dysfunction, leukotrienes, and lymphedema. Annu Rev Physiol. (2018) 80:49–70. doi: 10.1146/annurev-physiol-022516-034008 29029593 PMC6434710

[7] Zhang L An R Zhang G Gao L Shen W Wang Y . The clinical characteristics of systemic lupus erythematosus-related thoracic duct obstruction: a retrospective case-control study from China. Clin Rheumatol. (2024) 43:3689–97. doi: 10.1007/s10067-024-07157-2 39400757

[8] Yang L Sun Y Wen Z Wang R Yue Y . Systemic lupus erythematosus and rheumatoid arthritis with lymphatic system involvement: a study based on noncontrast MR lymphangiography and 99TCm-DX lymphoscintigraphy. BMC Med Imaging. (2026) 26:184. doi: 10.1186/s12880-026-02247-w 41764432 PMC13067621

[9] Cho Y Anderson EW Guevara SJ Miyara SJ Maria N Metz CN . Diagnostic dilemma of paraneoplastic rheumatic disorders: case series and narrative review. Cureus. (2021) 13:e19993. doi: 10.7759/cureus.19993 34984145 PMC8715838

[10] Iskra I Bogović Crnčić T Girotto N . Lymphoscintigraphy in lymphedema. Acta Clin Croat. (2024) 63:647–55. doi: 10.20471/acc.2024.63.03-04.24 41050262 PMC12490469

[11] Pappalardo M Cheng MH . Lymphoscintigraphy for the diagnosis of extremity lymphedema: current controversies regarding prtocol, interpretation, and clinical application. J Surg Oncol. (2020) 121:37–47. doi: 10.1002/iso.25526 31209893

[12] Basavalingu D Elojeimy S Mercer MK Itani M Revels JW Blacklock LC . Nuclear medicine lymphoscintigraphy: applications and technical overview. Radiographics. (2025) 45:e240207. doi: 10.1148/rg.240207 40569836

[13] de Souza PK Amorim RO Sousa LS Batista MD . Dermatological manifestations of hematologic neoplasms. Part II: nonspecific skin lesions/paraneoplastic diseases. Bras Dermatol. (2023) 98:141–58. doi: 10.1016/j.abd.2022.08.005 36682966 PMC9984718

[14] Clark J Kowlessur M VanderVeer E Shojania K Au S Kim H . Hematologic malignancies masquerading as rheumatologic diseases: a case series and review. Med (Baltimore). (2025) 104:e42251. doi: 10.1097/MD.0000000000042251 40295291 PMC12039978

[15] Ogawa-Momohara M Muro Y Mitsuma T Katayama M Yanaba K Nara M . Strong correlation between cancer progression and anti-transcription intermediary factor 1γ antibodies in dermatomyositis patients. Clin Exp Rheumatol. (2018) 36:990–5. 29745874

[16] Oldroyd AGS Allard AB Callen JP Chinoy H Chung L Fiorentino D . A systematic review and meta-analysis to inform cancer screening guidelines in idiopathic inflammatory myopathies. Rheumatol (Oxford). (2021) 60:2615–28. doi: 10.1093/rheumatology/keab166 33599244 PMC8213426

[17] Muro Y Nishida K Yamashita Y Koizumi H Takeichi T Satoh M . Comment on: favourable complete remission of anti-OJ antibody-positive myositis after lung cancer resection. Rheumatology. (2022) 61:e232–4. doi: 10.1093/rheumatology/keac170 35325040

[18] Shimada K . Recent advances in understanding and management of intravascular large B-cell lymphoma. Jpn J Clin Oncol. (2026) 55:1201–10. doi: 10.1093/jjco/hyaf112 40632023 PMC12596732

